# Female Nurses in the Male Wards of Mental Hospitals in Scotland
*Read in London on February 17, 1916, at the general meeting of the Medico-Psychological Association.


**Published:** 1916-02-26

**Authors:** George M. Robertson

**Affiliations:** Physician Superintendent of the Royal Edinburgh Asylum, Morningside, and Lecturer on Mental Diseases in the University of Edinburgh


### FEMALE NURSES IN THE MALE WARDS OF MENTAL HOSPITALS IN SCOTLAND.*

By GEORGE M. ROBERTSON, M.D., F.R.C.P. Edin.,
Physician Superintendent of the Royal Edinburgh Asylum, Morningside, and Lecturer on Mental Diseases in the University of Edinburgh.

Many of us have now grown so accustomed to female nursing and value it so highly that, on contemplating the subject, the question that comes most readily to our minds is, Why were women not always employed? To others who only know the mental hospital as it now is, with its carefully designed accommodation and its comfort, its good order and discipline, its skilful and intelligent nursing staff, its prevailing atmosphere of consideration for the patients and their medical care, the question also seems a very obvious one. But the mental hospitals of to-day are not less remote from the madhouses of 125 years ago than the period we live in from the Bronze Age, though we inherit many archaic traditions and practices from them. The madhouses at the end of the eighteenth and the beginning of the nineteenth century were not hospitals: they were prisons for the safe custody of a dangerous class. Little wonder, then, that the methods they adopted were those of the prison, that "keepers" alone were employed on the male side, and that women were rigidly excluded from it. The modern mental hospital can justly claim to be classed with other medical institutions, but, if so, it should fall into line with them by discarding an anachronism and by making use of women's mothering instincts and natural gifts for the nursing and care of male patients, as other hospitals have done. There rests, it seems to me, a heavy responsibility upon those who now fail to do so.

### Auxiliary Female Care.

The story of the introduction of women's help on the male side of asylums forms an instructive chapter in the history of the care of the insane, but it is only possible here to refer to the most important landmarks. Judging by the inertia in their employment that still exists in many quarters, although in a neighbouring country its success is an established fact, the man who first employed women in this way must have been of a very independent and original spirit. He was no less a person than Dr. Samuel Hitch, the founder of the Medico-Psychological Association. He introduced this system into the Gloucester General Lunatic Asylum in the very same year that he founded the Medico-Psychological Association—namely, 1841—and could he have survived till to-day to see the development attained by the twin offspring of his mind, he would have much reason to be proud of both of them. Dr. Hitch employed the wives of his married charge attendants to help their husbands in the male wards, and I was informed by his widow that it was because of the harsh manner in which the male patients were then treated by the attendants that he was induced to take this step. This statement is confirmed by the minutes of the asylum, which I was permitted to see, in which it is recorded that this woman's husband had charge of the refractory ward. Dr. Hitch's lead was followed by many English asylums during the next forty years.

Another step in advance was taken in 1883 by Dr. R. M. Bucke, of the London Asylum in Canada, who employed widows of good character in the male wards. Dr. Bucke was one of the most striking personalities of the American Medico-Psychological Association, and this experiment in his hands was a complete success.

A third step was taken by Sir Thomas Clouston, who had adopted Dr. Hitch's system by placing a married couple in charge of the Male Hospital at Morningside. The husband then died, and in 1890 he appointed the widow in full charge, with the male attendants under her authority. He permitted her to engage the services of two ordinary asylum nurses to assist her, and occasionally female patients would help as well.

These three methods for the employment of women on the male side—namely, Dr. Hitch's, Dr. Bucke's, and Dr. Clouston's—illustrate what I regard as the phase of *Auxiliary Female Care.* Its defect lies in the fact that the nurses were few in number, and only assisted the male attendants, and the bulk of the nursing, even in those wards in which they were employed, continued to be done by the male attendants. It would appear that no danger was apprehended to these women from the violence of the patients, but the selection of wives or widows of good character indicates that the risk of misconduct was recognised and thus guarded against.

### Entire Female Nursing.

The first step towards the system by which a group of male patients is entirely nursed by women was taken by Dr. Turnbull in the Fife and Kinross Asylum in 1896. He placed a ward containing thirty male hospital patients, by day only, entirely under the charge of female nurses. Owing to the construction of the hospital very efficient supervision was capable of being exercised over these nurses by the matron and the charge nurse of the female hospital. The proximity to the other male wards also enabled male help to be immediately summoned if necessary. The Scottish Commissioners in Lunacy at once realised that Dr. Turnbull's innovation constituted an important new departure and a great advance on anything that had been attempted before. Acting on the advice of Sir John Sibbald, who thought very highly of it, a similar arrangement was soon after introduced into the Glasgow (Gartloch), Lanark, and Perth District Asylums. In the year 1900, at the Stirling District Asylum, I placed a group of male patients, by night as well as by day, under the charge of female nurses, thus for the first time frankly handing over the entire care and nursing of insane male patients to women alone during the whole twenty-four hours.

These arrangements just described may very appropriately be called the Scottish system of entire female nursing, for its special features were not only developed in the Scottish asylums, but it has been very extensively adopted by them for nearly a generation. It is a totally different thing in practice from the system I have called that of auxiliary female care, in which a few women assisted the male attendants. All who have had great experience of women nurses in male wards agree in saying that they are infinitely more useful if placed in sole

* Read in London on February 17, 1916, at the general meeting of the Medico-Psychological Association.

charge of a group, and they much prefer it themselves. The patients benefit more certainly from their ministrations, for the Scottish system provides a guarantee that they must be nursed by women. Under the system of auxiliary female care there is a division of labour in the ward, and the nursing may be done by the men and the cleaning-up and household duties by the women. The danger of misconduct, which has been already referred to, decreases as the number of nurses employed becomes greater, and is least when the care of a whole ward is handed over entirely to women. It must be remembered too, that supervision and discipline in the asylums of the early and mid Victorian period, when the system of auxiliary female care was employed, were not so perfect then as they are now. What with the advent of efficient supervision and of the good class of men and women now engaged in asylum work, difficulties of this nature can be overcome, and need not be feared in a modern asylum.

